# Editorial: Alveolar Bone: A Pivotal Role in Periodontal Disease Pathobiology and Treatment

**DOI:** 10.3389/fphys.2022.889111

**Published:** 2022-04-12

**Authors:** Fani Anagnostou, Beatriz Castaneda, Frédéric Lézot, Petros Papagerakis

**Affiliations:** ^1^ Service of Odontology, Hospital Pitié-Salpêtrière AP-HP, Paris, France; ^2^ B3OA, CNRS UMR 7052, INSERM U1271, ENVA, University of Paris, Paris, France; ^3^ Laboratory of Childhood Genetic Diseases, INSERM UMR933, Hospital Armand Trousseau AP-HP, Sorbonne University, Paris, France; ^4^ College of Dentistry, University of Saskatchewan, Saskatoon, SK, Canada

**Keywords:** alveolar bone, periodontal disease, Pathobiology, treatment, tooth, osseointegration

The periodontal disease (PD) is a complex disease of multifactorial etiology, mainly characterized by a gingival inflammation and an alveolar bone destruction (Albandar, 2005). The major etiological factor appears to be the chronic exposure to germs of the oral bacterial flora (Haffajee et al., 2008) but bacteria do not cause the destruction of periodontal tissues by themselves. Bacteria stimulate an inflammatory immune response accountable for such destruction. So, a cascade of cellular and biochemical events take their place in the pathological progression leading to PD with the final disruption of connective and bone tissues homeostasis (Hajishengallis and Sahingur, 2014). The local inflammatory response may be either attenuated or inversely amplified by several risk factors from genetic or environmental origins as well as patient lifestyle (Albandar, 2005; Nikolopoulos et al., 2008).

The aim of the present Research Topic was to assemble manuscripts addressing the questions relating to the alveolar bone physiopathology in the PD from the pathogenesis to the therapeutic approaches. In fine, this Research Topic assembles nine original research manuscripts and one mini-review manuscript.

Regarding the alveolar bone physiopathology, the manuscript by Kwack et al. and collaborators demonstrates the presence of a myeloid cell heterogeneity profile in the bone marrow of the alveolar bone that is distinct from what is observed in the bone marrow of long bones. Indeed, a reduced myeloid-derived suppressor cell population was reported that, however, evidences an increased immunosuppressive activity. In parallel, an increased B lymphocyte population was evidenced. Interestingly, to put in perspective to such B lymphocyte population increase, the manuscript by Settem et al. and collaborators, using an elegant model of conditional invalidation in mouse, demonstrated that RANKL expression by B lymphocyte contributes to the alveolar bone loss characteristic of the PD. The study presented by Yamada et al. and collaborators reports, using also a mouse model, that the Loeys-Dietz syndrome corresponding to alterations of the TGFβ signaling favors the occurrence and progression of periodontitis. This increased susceptibility associated to TGFβ signaling dysfunction may be linked to the critical part played by the TGFβ signaling in the proliferation and differentiation of osteogenic progenitor cells during the postnatal alveolar bone formation presented here by Xu et al. and collaborators. This study, with TGFβ-RII conditionally invalidated mice, at early or late stages of the osteoblast differentiation using the CRE expression driven respectively by Gli1 and Col1a1 promotors, outlines the requirement of the TGFβ signaling for a functional alveolo-periodontal-dental complex formation.

Regarding the part of the periodontal ligament compartment in the PD, the manuscript by Li et al. and collaborators evidenced that epithelial cell rests of Malassez provide a favorable microenvironment to enforce the osteogenic potential of human periodontal ligament stem cells that was impaired in the periodontitis pathological or aged context. The manuscript by Mayr et al. and collaborators demonstrated that autophagy induces the expression of IL-6 by human periodontal ligament fibroblasts in response to mechanical load and overload with impact on the osteoclastogenesis and consequently the alveolar bone loss. Interestingly, Aljohani et al. and collaborators evidenced that the Methylsulfonylmethane, a naturally occurring anti-inflammatory compound, was able to increase the alveolar bone density in aged mice and so it may be of use as complementary treatment to improve bone loss-associated diseases, including PD. Finally, the mini-review presented by Syazana et al. and collaborators concerns another promising approach for PD treatment, up to now misestimated, the salivary exosomes. These exosomes provide miRNA, proteins, lipids and signaling molecules that may enable to protect the periodontal tissues and maintain the alveolar bone in the inflammatory context of PD.

The last but not least two manuscripts are relative to the osseointegration which is a key process of the restoration of a functional alveolo-periodontal-dental complex following the loss of teeth frequently due to the PD. The manuscript by Wang et al. and collaborators evidenced that high-altitude induced hypoxia has a negative effect on early osseointegration of titanium implants but such effect can be mitigated by the use of implants with rough surfaces, found to increase the expression osteogenesis-related genes in MG-63 cells under hypoxic conditions. The manuscript by Huang et al. and collaborators reported that Adipodon, an oral active synthetic small molecule with biological functions similar to adiponectin, was able to improve bone micro-architecture and to enhance the osseointegration in the diabetes mellitus-impaired bone microenvironment, which may open new perspectives for the treatment of PD and its consequences in diabetic patients.

To conclude this Research Topic underlines the dynamism of the researches on the periodontal disease with progresses in our understanding of the molecular and cellular mechanisms subjacent to the disease initiation and progression, but also in the therapeutic management of the disease whatever its stage of development. In perspective, there is no doubt that on the one hand an earlier and efficient management of the periodontal disease will be possible thanks to actual breakthrough in the molecular deciphering of this multifactorial pathology, and on the other hand that new treatments and therapeutic approaches will be available for patients in more advanced stages of the pathology.

The guest Editors want to thank all the authors for sharing their highly interesting results in this topic and all the Reviewers that kingly give their time and make this research topic possible.
